# Genetic affinities among the lower castes and tribal groups of India: inference from Y chromosome and mitochondrial DNA

**DOI:** 10.1186/1471-2156-7-42

**Published:** 2006-08-07

**Authors:** Ismail Thanseem, Kumarasamy Thangaraj, Gyaneshwer Chaubey, Vijay Kumar Singh, Lakkakula VKS Bhaskar, B Mohan Reddy, Alla G Reddy, Lalji Singh

**Affiliations:** 1 Centre for Cellular and Molecular Biology, Uppal Road, Hyderabad- 500 007, India; 2 Estonian Biocentre, Riia, 23, Tartu- 51010, Estonia; 3 Biological Anthropology Unit, Indian Statistical Research Institute, Habsiguda, Hyderabad, India

## Abstract

**Background:**

India is a country with enormous social and cultural diversity due to its positioning on the crossroads of many historic and pre-historic human migrations. The hierarchical caste system in the Hindu society dominates the social structure of the Indian populations. The origin of the caste system in India is a matter of debate with many linguists and anthropologists suggesting that it began with the arrival of Indo-European speakers from Central Asia about 3500 years ago. Previous genetic studies based on Indian populations failed to achieve a consensus in this regard. We analysed the Y-chromosome and mitochondrial DNA of three tribal populations of southern India, compared the results with available data from the Indian subcontinent and tried to reconstruct the evolutionary history of Indian caste and tribal populations.

**Results:**

No significant difference was observed in the mitochondrial DNA between Indian tribal and caste populations, except for the presence of a higher frequency of west Eurasian-specific haplogroups in the higher castes, mostly in the north western part of India. On the other hand, the study of the Indian Y lineages revealed distinct distribution patterns among caste and tribal populations. The paternal lineages of Indian lower castes showed significantly closer affinity to the tribal populations than to the upper castes. The frequencies of deep-rooted Y haplogroups such as M89, M52, and M95 were higher in the lower castes and tribes, compared to the upper castes.

**Conclusion:**

The present study suggests that the vast majority (>98%) of the Indian maternal gene pool, consisting of Indio-European and Dravidian speakers, is genetically more or less uniform. Invasions after the late Pleistocene settlement might have been mostly male-mediated. However, Y-SNP data provides compelling genetic evidence for a tribal origin of the lower caste populations in the subcontinent. Lower caste groups might have originated with the hierarchical divisions that arose within the tribal groups with the spread of Neolithic agriculturalists, much earlier than the arrival of Aryan speakers. The Indo-Europeans established themselves as upper castes among this already developed caste-like class structure within the tribes.

## Background

"Out-of-Africa" hypothesis suggests that the anatomically modern humans originated in Africa about 160,000 – 150,000 years ago, and then spread outward, completely replacing the local archaic hominid populations outside Africa. India has served as a major corridor for the dispersal of modern humans out of Africa, owing to the positioning of the Indian Peninsula at the crossroads of Africa, the Pacific and the West and East Eurasia. The enormous cultural, linguistic and genetic diversity of the more than one billion people living in the contemporary ethnic India can be attributed to this. The Indian society and culture might have been affected by multiple waves of migration and gene flow that occurred in the historic and pre-historic times [1]. The first among this is the ancient Paleolithic migration by the modern humans during their initial colonization of Eurasia. This is followed by the early Neolithic migration, probably of proto-Dravidian speakers, from the eastern horn of the Fertile Crescent. The Indo-European speakers, who might have arrived ~3,500 years ago, are the third potential source of Indian gene pool. The Austro-Asiatic and Tibeto-Burman speakers with ties to East/Southeast Asia form the fourth major contributors. The most recent conquerors from Central Asia and the colonizers from Europe might also have added to this ethnic multiplicity.

The social structure of the Indian population is dominated by the hierarchical Hindu caste system. There are 4,635 well-defined endogamous populations in India, which are culturally stratified as tribes and non-tribes. The 532 tribal communities, who are supposed to be the aboriginal inhabitants of the sub-continent, constitute 7.76% of the total population (Indian Census – 2001). The origin of caste system in India is a matter of debate. Previous genetic studies on Indian castes and tribes failed to achieve a consensus on Indian origins and affinities. A few studies reported closer affinity of Indian castes with either the Europeans or the Asians. Studies of Bamshad et al [2] and Basu et al [3]support the genetic differentiation of caste and tribal populations, and the North Indian invasion of Indo-European speaking nomads, pushing the Dravidian tribes to southern peninsula. On the other hand, Kivisild et al [4] suggest that Indian tribal and caste populations derived largely from the same genetic heritage of Pleistocene southern and western Asians, receiving limited gene flow from external regions since Holocene. Further, Cordaux et al [5] reports that the paternal lineages of Indian castes are more closely related to the Central Asians than to the Indian tribal groups, thereby supporting the view that Indian caste groups are primarily the descendents of the Indo-European migrants. More studies are required for a better understanding of the genetic structure of the diverse Indian populations, where many questions remain unanswered. In the present study, mtDNA and Y chromosome of three different tribal populations of Andhra Pradesh (AP), South India, were analyzed. On comparing the results with available data, we were able to reconstruct the evolutionay history of Indian caste and tribal populations, by providing a comprehensive picture of their genetic structure.

## Results and discussion

### Mitochondrial DNA variation

The sequence data corresponding to nucleotide positions 15927 – 16550 [revised Cambridge Reference Sequence (rCRS)] [6] that includes the HVR I region was obtained from 347 individuals belonging to the three tribal populations. Insertions were observed at two positions (16169_16170insC, 16262_16263insT). Nucleotide substitutions were observed at 120 sites, defining 149 HVR I motifs. Seventy haplotypes were observed among Pardhan, 53 among Naikpod and 48 among Andh tribes. A total of 131 (76.5%) unique haplotypes were observed; 56 (80%) in Pardhan, 37 (70%) in Naikpod and 38 (79%) in Andh. Only two HVR I motifs were found to be shared among all the three populations; 10 haplotypes were shared between Pardhan and Naikpod, four between Pardhan and Andh and six between Naikpod and Andh. At the individual level, 43% of haplotypes were shared by two or more individuals, 75% of this being within the same population.

### Demographic expansion of the populations

Based on AMOVA, the variation among studied populations was only 2.1%, while the remaining 97.9% variation was within populations. The number of haplotypes, haplotype diversity, nucleotide diversity, mean number of mismatches, Fu's *Fs *statistic values, raggedness index (*r*), expansion ages and initial effective population sizes of the three populations are summarized in Table 1. The demographic history of each population was examined by computing the pairwise difference distributions. Unimodel distribution curves were observed, which could be interpreted as signs of demographic expansion. Likewise, the raggedness index was found to be less than 0.02 in all the populations studied; values of *r *lower than 0.05 also suggest demographic expansions [7]. Negative values of *Fs *that differ significantly from zero, and the significant (*P *< 0.05) negative *D *values, further support a recent expansion.

**Table 1:** Diversity and demographic parameters deduced from mtDNA HVR I sequences in the tribal populations of AP

**Population**	***Na***	***Nb***	***N***_***D***_	***π***	***k***	***τ***	***θ***_*a*_	***r***	***Y***	***Ne***	**FS**	D*
Pardhan	193	70	0.972	0.011 ± 0.006	6.691 ± 3.170	4.829	2.644	0.010	38943.55	1066.13	-24.73	-1.683
Naikpod	88	53	0.978	0.008 ± 0.004	5.593 ± 2.712	5.720	0.329	0.009	46129.03	132.66	-25.333	-1.562
Andh	66	48	0.987	0.009 ± 0.005	6.288 ± 3.021	5.410	0.817	0.016	43629.03	329.44	-25.18	-1.687

*Na*- number of sequences; *Nb*- number of haplotype; *N*_*D *_-Haplotype diversity; *π *– nucleotide diversity; *k*- mean number of mismatches; *τ - *tau; *θ*_*a*- _initial theta; *r*- raggedness index; *Y*- expansion time in ybp (*A*x *τ/*2*μ*); *Ne*- effective population size; *μ *= mutation rate (0.00124 per site per generation); *A *= generation time (20 years); *Fs*- Fu's Fs statistics; *D*- Tajima's D; *P < 0.05.

### Mitochondrial haplogroups

The frequencies of various mtDNA haplogroups in the three tribal populations are summarized in Table 2; a total of 27 different haplogroups were observed [see Additional file 2]. Of the 347 sequences, 67% belongs to the haplogroup M and its subclades. This is consistent with the previous studies, suggesting the frequency of M in the Indian tribal groups is more than that in caste populations [8]. M3, which is defined by the motif 482-16126, was the most frequent sub-clad, and it accounted for 17% of the total M lineages. This was followed by M2 (15%) and other undefined M lineages (16%). Other Indian-specific M-clades like M6, was found in fairly good frequencies in the Pardhan tribe (17% of M); M25 constituted 9.5% of the total M lineages in Naikpods. A relatively high frequency of M18 (13% of M and 8.3% of the total), defined by the transversion at 16318, was observed in Pardhan, while it was absent in the other two populations.

**Table 2:** Frequency (percentage) of different mtDNA haplogroups in Pardhan, Naikpod and Andh tribal populations

	Populations			
		
Haplogroup	Pardhan	Naikpod	Andh	Total
M	7.25	13.64	10.61	9.51
M2	8.29	11.36	13.64	10.09
M3	10.36	14.77	10.61	11.53
M4	2.07	1.14	4.55	2.31
M5	8.81	14.77	4.55	9.51
M6	10.88	2.27	0.00	6.63
M18	8.29	0.00	0.00	4.61
M25	0.52	6.82	1.52	2.31
M30	1.55	2.27	1.52	1.73
M33	0.00	3.41	3.03	1.44
M35	0.52	0.00	6.06	1.44
M38	2.07	1.14	0.00	1.44
M39	0.00	0.00	12.12	2.31
M40	0.52	0.00	0.00	0.29
M41	3.11	0.00	0.00	1.73
W	0.00	0.00	1.52	0.29
R	5.70	11.36	1.52	6.34
R5	12.44	4.55	12.12	10.66
R6	1.04	0.00	3.03	1.15
R7	1.04	0.00	1.52	0.86
H14	0.00	3.41	0.00	0.86
J2	3.11	0.00	0.00	1.73
K	0.52	0.00	1.52	0.58
T	0.52	0.00	1.52	0.58
U2	5.70	1.14	6.06	4.61
U2a	2.07	7.95	0.00	3.17
U5	3.11	0.00	3.03	2.31

The newly defined Indian-specific mitochondrial sub-clad, M41 [9], was found in ~5% of the Pardhan M samples. This lineage was previously reported as an undefined M lineage found at a very low frequency in caste (Brahmin, Yadava and Mala) and tribal (Koya and Lambadi) populations of AP, but not anywhere else in India [4,10,11].

Macrohaplogroup N constituted 33% of the studied samples, and vast majority of them belonged to Indian-specific variants of the phylogenetic node R, including haplogroups R5, R6 and U2. The most frequent sub-clade of R was R5 (35% of total R), followed by U2 (25%). A new package for the Indian-specific mtDNA clades has been proposed by Metspalu et al [8] which includes deep-rooted lineages of M2, R5 and U2, since these constitute nearly 15% of the Indian mtDNAs, and being virtually absent in Eurasia. In the present study, this Indian package harbors 28.5% of all samples, much more than the Indian average; this is a genetic testimony for their ancient origins.

**Figure 1:**
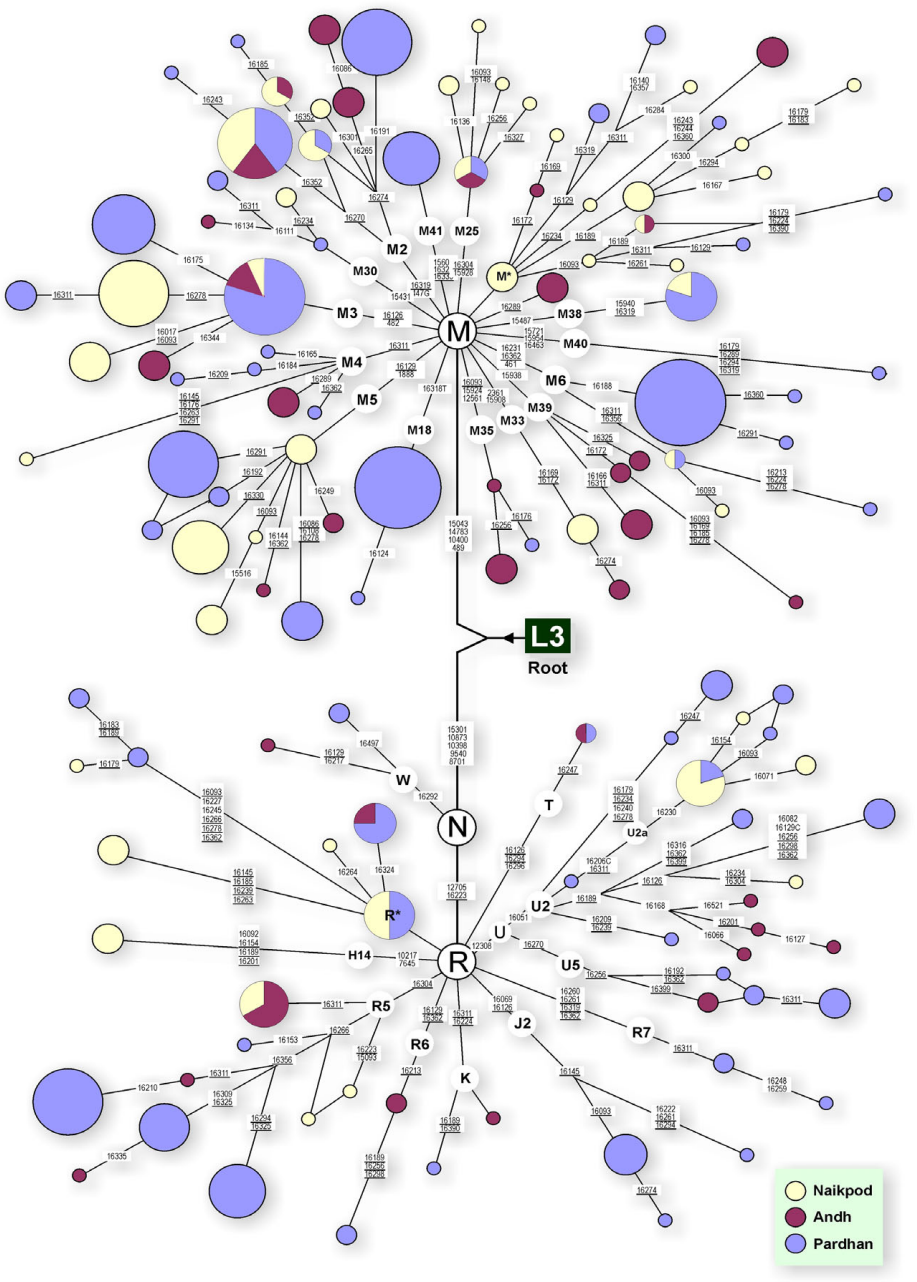
A network relating Pardhan, Naikpod and Andh haplotypes. Circle areas are proportional to the haplotype frequencies. Variant bases are numbered and shown along the links between haplotypes. Character change is specified only for transversions. Mutations in the HVR I region are only mentioned except that defines haplogroups. Variations at hyper-variable positions 16182, 16183 and 16517 are not shown

Median joining networks were constructed, showing the distribution of various "limbs" and "boughs" of the M and R "trunks", and a star-like topology (Figure 1). These star-like clusters reflect the demographic expansion of the studied populations.

### Genetic uniformity of Indian maternal lineages

The present HVR I data from the three populations, together with the 10 previously reported data from South Indian tribal populations, were compared with that of caste populations published before [4,8,12,10,13] (Table 3). AMOVA revealed that the tribal populations showed a variation of 6.38 % among themselves. Variation between the tribal groups and lower castes was found to be only 0.5%. A higher diversity was observed (0.93%) between the tribal populations and upper castes. Significant difference was not observed when the upper caste populations were split according to their linguistic affiliation. Difference between upper and lower castes was also found to be very low (0.46%).

**Table 3:** Populations included in the study for the comparison of HVR I data.

**Group**	**Populations**	**Sample size**	**Reference**
Indian Upper Castes	Andhra Brahmin	39	[10]
	Karnataka Brahmin	47	[12]
	Maharashtra Brahmin	58	[8]
	Punjab Brahmin	26	[8]
	Uttar Pradesh Brahmin	25	[8]
	West Bengal Brahmin	39	[8]
	Kshatriya	34	[8]
	Rajput	35	[8]

Indian Lower Castes	Kapu	52	[10]
	Madiga	26	[10]
	Relli	20	[10]
	Yadhava	47	[10]
	Mala	24	[10]

Indian Tribes	Pardhan	193	Present study
	Andh	66	Present study
	Naikpod	88	Present study
	Chenchu	94	[4]
	Koya	81	[4]
	Lambadi	86	[4]
	Pardhi	42	[13]
	Thoti	39	[13]
	Koragas	53	[13]
	Kuruchian	46	[13]
	Mullukurunan	44	[13]
	Yerava	53	[13]
	Kota	25	[13]

Roychoudhury et al [14] had suggested that Indian populations were founded by a rather small number of females, possibly arriving on one of the early waves of out-of-Africa migration of modern humans; ethnic differentiation occurred subsequently, through demographic expansions and geographic dispersal. Lack of L3 mitochondrial lineages other than M and N in India, and in non-African mtDNAs in general, suggest that these earliest migrants might have already carried these two mtDNA ancestors [9]. The coalescence time of Indian M lineages was found to be older than that of most of the East Asian and Melanesian M clusters [15]. These results suggest that the Indian subcontinent was settled soon after the initial out-of-Africa expedition, and that there had been no complete extinction or replacement of the initial settlers [9]; rather it might have been restructured *in situ *by the major demographic episodes of the past, and by the relatively minor gene flow due to the recent invasions from both the West and the East [8]. In view of the stringent mating practices imposed by the caste system in India, our present study strongly suggests a common maternal ancestry, rather than an extensive recent gene flow between the caste and tribal populations. However, the presence of western Eurasian-specific mtDNA haplogroups like HV, TJ and N1 in comparatively higher frequencies among upper castes, is suggestive of recent maternal gene flow. They are likely to represent a relatively low-intensity, long-lasting admixture at the western border regions, as well as migrations during the last 1000 years before present (ybp) [11].

### Y SNP analysis- relationship of lower castes with the tribes

**Figure 2:**
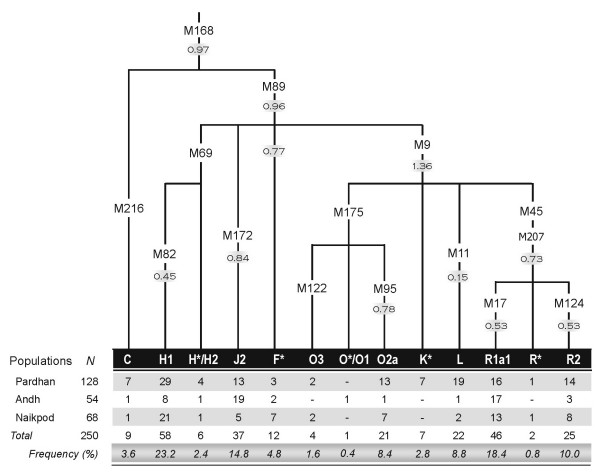
Y chromosomal haplogroups and their frequencies (%)in three South Indian tribal populations. Haplogroup defining markers and their background average variance of 6 STR loci are shown along the branches of the tree.

A Y-chromosomal haplogroup tree, based on 16 biallelic markers, of 250 male samples from the three tribal populations was constructed. The haplogroup frequencies observed, and their background average variance of 6 Y-STR (short tandem repeat) loci are shown in Figure 2.

AMOVA analysis revealed 2.77% variation among the three tribal populations studied. They showed 2.18% variation, when compared with the 8 South Indian tribal populations studied earlier. When Andh samples were omitted from the analysis, the other two populations showed more closeness to the other Indian tribes, with 1.72% variation. Interestingly, the variation between the studied tribal populations and 13 Indian caste populations (Table 4) was only 1.14%. When all the available data were combined (11 tribes and 13 castes) the variance between the groups became 2.85% (north-east Indian tribal data omitted). This is a remarkably lower value than the earlier report of Cordaux et al [13], where they found 13% variation between the Indian tribal and caste groups. In this study, however, we omitted populations with samples size less than 20, and those for which all biallelic polymorphisms were not typed. A total of 508 tribal and 901 caste samples were included in the analysis; this forms the broadest dataset of Indian Y chromosomes, so far [see Additional file 1].

**Table 4:** Frequencies of different Y biallelic markers among the upper caste, lower caste and tribal populations of India. (Total share in percentage is given in brackets)

			Y SNP based major haplogr oups					
			
**Group**	**Populations**	***N***	L (M11, M20, M27)	J (M172, M67)	R (M207, M17, M124)	F* (M89)	H (M52, M69, M82)	O (M175 M122, M95)
Indian Upper Castes	Brahmin^*b*^	403	0.16 (39)	0.14 (47)	0.44 (48)	0.05 (8)	0.09 (14)	0.02 (12)
	Konka^*d*^							
	Khathriya^*b*^							
	Sourashtra^*e*^							
	Punjab^*d*^							
	Vizag- Brahmins^*f*^							
	Peruru- Brahmins^*f*^							
	Kammas^*f*^							

Indian Lower Castes	Bahelia^*b*^	374	0.18 (44)	0.07 (23)	0.22 (24)	0.13 (52)	0.25 (39)	0.06 (35)
	Kallar^*e*^							
	Lower							
	Manghi^*b*^							
	Telega^*b*^							
	Yadhva^*e*^							

Indian Tribes	Andh^*a*^	508	0.07 (17)	0.09 (30)	0.26 (28)	0.10 (40)	0.30 (47)	0.09 (53)
	Bagata^*f*^							
	Chenchu^*d*^							
	Koraga^*c*^							
	Koya^*d*^							
	Lambadi^*d*^							
	Naikpod^*a*^							
	Pardhan^*a*^							
	Paroja^*f*^							
	Valmiki^*f*^							
	Yerava^*c*^							

*a*- present study, *b*- unpublished data, c- [5], *d*- [4], *e*- [44], *f *– [45]. *N *= total number of samples

**Figure 3:**
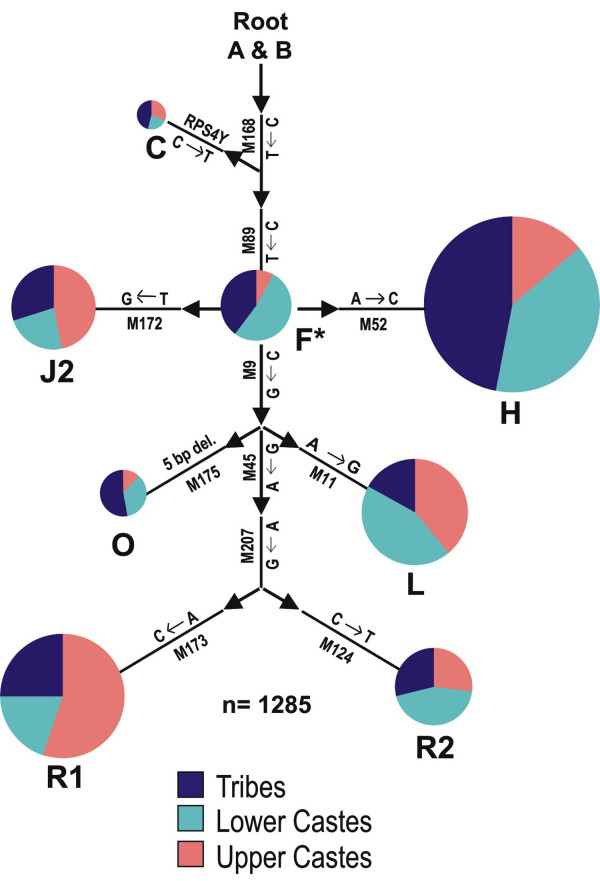
Distribution of major Y-SNP haplogroups among the tribal, lower caste and upper caste populations of India.

The frequencies of major Y-SNP haplogroups in the different Indian populations are given in Table 4. The most frequent haplogroup among the Indian upper castes belongs to R lineages (R*, R1 and R2); together, these account for 44% of the upper caste Y-chromosomes. Haplogroup H was the most frequent Y lineage in both the lower castes and tribal populations, with frequencies of 0.25 and 0.30, respectively. The Indian Y-SNP tree (Figure 3) shows that the distribution pattern of the major Y lineages is similar in tribal and lower caste populations, and is distinct from the upper castes.

Although many other nations are known for social discrimination, perhaps, nowhere else in the world has inequality been so elaborately deep rooted as in the Indian institution of caste, even though it has undergone significant changes since independence. An interesting observation in the present study is that the available Y-SNP data from 374 individuals belonging to five lower caste populations from different geographic regions with various linguistic affiliations, show only 1% variation with the South Indian Dravidian tribal groups. The lower caste shows more similarity with the tribal groups than with the upper caste populations (4.72% difference between the upper and lower castes). This is suggestive of a tribal origin for the Indian lower castes. Geography does not seem to have affected this association of the tribal groups with the lower castes, since there was no significant difference in the AMOVA value when the lower castes were grouped as northern and southern, based on their geographic locations. At the same time, significant variation (6.17%) was observed between upper castes and tribal groups (Table 5). However, variation of Dravidian tribal groups with Dravidian higher castes was found to be lower (4.4%) than that with Indo-European speaking north Indian higher castes (8.1%). The Indian lower castes that constitute around 68% of the total population are more paternally related with the tribal community, who are believed to be the original inhabitants of the subcontinent, following the initial settlement during late Pleistocene.

**Table 5:** Analyses of molecular variance based on mtDNA HVR I sequence and 16 Y-biallelic markers between the population groups of India

	Percentage of Variance	
	
Population Groups	mtDNA HVR I	Y-Chromosome
Among the tribal groups	6.38	2.18
Between tribes and lower castes	0.50	1.0
Between tribes and upper castes	0.93	6.17
Between lower and upper castes	0.46	4.72

The Y-SNP markers that are likely to have an Indian origin [F* (M89), H (M52), and O (M95)], as suggested earlier [5], were found in high frequency (Table 4), both in the tribes and in the lower castes. Around 89% of the samples with these clads belongs to either the tribes or the lower castes. Previously, it was reported that M52 should not be considered a tribal marker, as its frequency is concentrated regionally around AP [4]. However, in our study of 250 tribal samples from AP, its frequency was 0.25, while for 112 samples from two lower caste populations from Madhya Pradesh and Jharkhand, the frequency was found to be 0.36. Hence, it is a lower caste/tribal marker, rather than a tribal marker alone, and is widely distributed. The origin of M52 within the subcontinent, immediately after late Pleistocene settlement, cannot be ruled out, since it is the major Y lineage of more than 85% of the hierarchical Hindu caste system, and spread throughout the country except the North East. Limited presence of this clad in Central Asia and in European gypsy populations [16] may be due to the recent back migrations, and there are several theories about their Indian ancestry [17]. However, the relatively low STR variance of H haplogroup in comparison with the other Indian haplogroups (Figure 2) is slightly unexpected, and may need further investigations with additional markers and samples.

### M 95- genetic footprints of earliest settlers?

The M95 lineage (O2) is a predominantly Southeast Asian haplogroup among the Austro Asiatic speakers [18]. In our analysis, M95 mutation was detected in 6.3% of the Indian samples with highest frequency in lower castes and tribes. A high frequency of M95 is also expected from the Indian Austro-Asiatic speaking populations, like their Southeast Asian counterparts, who were hypothesized to be the earliest settlers of the Indian sub-continent [19]. Of the two major groups of Austro-Asiatic tribes in the Indian subcontinent, the Mundari speakers are proposed to be non-Asian/African in origin, who arrived in the subcontinent taking a southern coastal route [20]. Hence, it is reasonable to assume that the higher frequency of M95 in South Indian tribal populations is the footprints of these initial settlers, who already carried the defining mutation, and later spread to Southeast Asia. The higher STR variance observed among the M95 samples of the present study also supports their early settlement in the Indian sub-continent. Interestingly, the TMRCA (time to most recent common ancestory) of the Southeast Asian M95 is estimated to be only ~8000 years, with a star of population expansion ~4,400 years ago [18].

### The Neolithic contributions

The J172 clad was observed in about 10% of the Indian populations, with almost half of them belonging to upper castes; its frequency was much lower among the tribes (0.06) and lower castes (0.07). The macrohaplogroup J is proposed to have arisen in the Levant, and perhaps, associated with the spread of Neolithic culture. However, more archeological, linguistic and genetic evidences are necessary to hypothesize that M172 is a part of 'Neolithic genes' that invaded the Indian subcontinent with Dravidian agriculturalists, since we observed very high STR diversity for J haplogroup in the Dravidian tribal populations.

Frequency of haplogroup L- (M11/M20), which is also proposed to be associated with the expansion of farming, was 13.7%, with the highest occurrences in caste populations. A similar frequency of L lineage has previously been reported from Pakistan [21]. An M27 mutation that defined the subclad L1 was found in all the L-M11 samples in the present study. This is in accordance with the previous studies that M27 characterizes the Indian and Pakistani lineages, which is absent in their Turkish counterparts [22]. This result, together with the differences in STR nodal haplotypes of the L clad between the Caucasus and Indian populations [4], and matches in the six STR loci typed between Turkish and Armenians [22], lead to the assumption that the Indian and Pakistani L lineages might have originated from a distinct founder population. This view is supported by the much lower STR variance of the L haplogroups compared with the other Indian Y-lineages, observed in the present study.

### Preexistence of R lineages in the subcontinent

The sister clads; R1a1 (M17) and R2 (M124) of the M207 lineage together form the largest Y haplogroup lineage in India, with a frequency of 0.32. They are present in substantial frequencies throughout the subcontinent, irrespective of the regional and linguistic barriers. The haplogroup R-M17 also has a wide geographic distribution in Europe, West Asia and the Middle East, with highest frequencies in Eastern European populations [23]. It is proposed to be originated in the Eurasian Steppes, north of the Black and Caspian seas, in a population of the Kurgan culture known for the domestication of horse, ~3500 ybp [23], and widely been regarded as a marker for the male-mediated Indo-Aryan invasion of Indian subcontinent. However, these observations were contradicted by the higher STR variations observed in the Indian M17 and M124 samples, compared with the European and Central Asian populations, suggesting a much deeper time depth for the origin of the Indian M17 lineages. In the present study, it was observed that the R lineages were successfully penetrated to high frequencies (0.26) in the South Indian tribal populations, a testimony for its arrival in the peninsula much before the recent migrations of Indo-European pastoralists from Central Asia. In a recent study, Sengupta et al [24] observed higher microsatellite variance, and clustering together of Indian M17 lineages compared with the Middle East and Europe. They proposed that it is an early invasion of M17 during the Holocene expansion that contributed to the tribal gene pool in India, than a recent gene flow from Indo-European nomads. However, we found that its frequency is much higher in upper castes (0.44) compared to that of the lower caste (0.22) and tribal groups (0.26). This uneven distribution pattern shows that the recent immigrations from Central Asia also contributed undoubtedly to a pre-existing gene pool.

### Lower castes: uplifted tribal groups?

The origin of the caste system in India remains an enigma, although several theories suggested that it began with the arrival of Aryans [25]. However, many linguistics and anthropologists argue that caste system prevailed in India even before the entry of Aryan speakers [26]. Many castes are known to have tribal origins, as evidenced from various totemic features that manifest themselves in these caste groups [27]. The caste system might have developed as a class structure from within the tribes, with the spread of Neolithic agriculturalists as suggested by Majumder [28]. Kosambi [27] also pointed out that the knowledge and ownership of the means of food production might have created hierarchical divisions within the tribal societies. The origin of present day lower castes should be traced back to this period, rather than the recent Aryan migrations and admixture. Molecular data from the present study can be considered as a genetic testimony in support of these viewpoints on the origin of caste system in India.

## Conclusion

Our study suggests that the vast majority (>98%) of the Indian maternal gene pool that consists of the Dravidian and Indo-European speakers is genetically more similar, and received only minor gene flow with the recent invasions from both the West and the East, since their initial late Pleistocene settlement. On the other hand, the Indian Y-chromosome lineages show obvious difference in their distribution pattern among the tribal and caste populations. However, the lower castes, (backward classes and scheduled castes, as per the Indian Constitution) show striking similarity with the Indian tribal populations. These groups, which constitute more than 85% of the hierarchical Hindu caste system, have the indigenous M52, M95 and M89, as their major Y lineages. This result suggests that the Indian lower castes are genetically more associated with the tribal populations, than to the higher castes, an evocative of their tribal origins. The presence of these native haplogroups in the Indo-European nomads, who arrived ~3500 ybp and established themselves as upper castes, might be due to the recent admixture with the local populations. The presence of the so called west/central Asian lineages like J2, R1 and R2 in most of the endogamous tribal populations, and its higher STR diversity indicates its presence in the sub-continent much before the arrival of the Indo-European pastoralists. In short, the impact of their arrival in the Indian sub-continent is rather social and political, than genetic.

## Methods

### DNA isolation

About 10 ml of blood samples from healthy unrelated individuals belonging to three tribal populations namely Pardhan (n = 193), Naikpod (88) and Andh (66) were collected from the northwestern region of Adilabad district of AP, southern India with their informed written consent with the help of the Tribal Welfare Department, Government of AP. DNA was isolated from the samples using the standard protocol [29].

### Amplification of mitochondrial DNA

The hyper-variable regions (HVR I and HVR II) and selected coding regions of the mtDNA were amplified from 10 ng of template DNA using 10 pM of each primer, 100 μM dNTPs, 1.5 mM MgCl_2 _and 1 U of *Taq *DNA polymerase. Generally, 35 cycles of reaction was performed with 30 sec denaturation at 94°C, 1 min annealing at 58°C and 2 min extension at 72°C. Annealing temperature and time were slightly modified for few sets of primers. The reactions were carried out in MJ Research thermal cycler (PTC-200).

### Y-chromosomal markers

Sixteen Y-chromosome biallelic polymorphic markers *viz *M89, M216, M9, M45, M82, M69, M170, M172, M11, M175, M95, M122, M207, M173, M17, and M124 were typed to construct the Y-chromosome phylogeny of the studied populations according to Y- Chromosome Consortium nomenclature [30]. The PCR cycles were set-up with an initial denaturation of 5 min at 95°C, followed by 30–35 cycles of 30 sec at 94°C, 30 sec at the primer-specific annealing temperature (52 – 60°C), and 45 sec. at 72°C, and final extension of 7 min at 72°C. Length variations at 6 Y-STR loci, DYS19, DYS389-1, DYS389-2, DYS390, DYS391 and DYS393, were typed using previously published primer sequences [31]. The multiplex PCR amplifications were performed in reaction volumes of 10.0 μl with 1U of AmpliTaq Gold^® ^DNA polymerase (Applied Biosystems, Foster City, CA), 10 mM Tris-HCl (pH 8.3), 50 mM KCl, 1.5 mM MgCl_2_, 250 μm dNTPs, 3.0 μm of each primer (forward primers are fluorescently labeled), and 10 ng of DNA template. Thermal cycling conditions were as follows: (1) 95°C for 10 min, (2) 28 cycles: 94°C for 1 min, 55°C for 1 min, 72°C for 1 min, (3) 60°C for 45 min, and (4) 25°C hold. The PCR amplicons along with GS500 LIZ size standard were analyzed using the ABI 3730 DNA Analyser (Applied Biosystems, Foster City, CA). The raw data were analyzed using the GeneMapper v3.7 software program (Applied Biosystems, Foster City, CA).

### Sequencing of the PCR products

PCR products were directly sequenced using BigDye™ Terminator cycle sequencing kit (Applied Biosystems) in ABI Prism 3730 DNA Analyzer following manufacture's protocol. The individual mtDNA sequences were judged against the rCRS [6] using AutoAssembler – *ver *2.1 (Applied Biosystems, Foster City, USA). The sequences were aligned using CLUSTAL X [32,33], and mutation data were scored with MEGA ver 3.1 [34,35]. Mitochondrial haplogroups were assigned to all samples according to Sun et al [36] and Thangaraj et al [9].

### Phylogenetic and statistical analyses

Data analyses for mtDNA sequences and Y-SNPs were performed using the ARLEQUIN software package [37,38]. Haplotype- and nucleotide- diversity and their standard deviations (SD); mismatch distributions, mean pairwise differences and their SD; Fu's *Fs *statistics [39] and associated *P*-values based on 1000 stimulated samples, raggedness index '*r'*, *F*st distances between pairs of populations and associated *P*-values based on 1000 permutations and Tajima's *D *value [40] were calculated. Analyses of molecular variance (AMOVA) were performed to evaluate the genetic structure of the populations; the significance of variance components tested with 10,000 permutations. Other statistical inferences, including initial theta (θ_*a*_) and values of *tau *(τ) were used to calculate effective population size (*Ne *= θ_*a*_/2μ) and population expansion age (*Y= A *x*τ*/2μ) [41]. An average mutation rate μ = 0.00124 per site per generation with an average generation time *A *= 20 years, was used for calculation. Median joining networks [42] were constructed with the help of Network 4.112 program [43] with default settings. Haplotype diversity and STR variance were calculated according to Kivisild et al [4].

## Authors' contributions

LS, KT and IT conceived the study and drafted the final manuscript. IT, GC, VKS and LVKSB performed the experiments and aligned the sequences. BMR participated in the design of the study. AGR contributed to the overall study design; all authors read, revised and approved the final manuscript.

## Supplementary Material

Additional File 1 Indian Y-SNP data. Y-chromosome biallelic polymorphism data of 1285 Indian samples are given. The Y-SNP and Y-STR haplotypes of the three populations analyzed are also provided. Click here for file

Additional File 2 Mitochondrial DNA mutation data. The data provided represent the mtDNA mutations at the HVR I region and selected coding regions from 347 tribal samples. Different haplogroups and its frequency are also given. Click here for file
